# Recent nanotheranostics applications for cancer therapy and diagnosis: A review

**DOI:** 10.1049/nbt2.12021

**Published:** 2021-02-14

**Authors:** Fahimeh Aminolroayaei, Daryoush Shahbazi‐Gahrouei, Saghar Shahbazi‐Gahrouei, Naser Rasouli

**Affiliations:** ^1^ Department of Medical Physics School of Medicine Isfahan University of Medical Sciences Isfahan Iran; ^2^ School of Medicine Isfahan University of Medical Sciences Isfahan Iran

## Abstract

Nanotheranostics has attracted much attention due to its widespread application in molecular imaging and cancer therapy. Molecular imaging using nanoparticles has attracted special attention in the diagnosis of cancer at early stages. With the progress made in nanotheranostics, studying drug release, accumulation in the target tissue, biodistribution, and treatment effectiveness are other important factors. However, according to the studies conducted in this regard, each nanoparticle has some advantages and limitations that should be examined and then used in clinical applications. The main goal of this review is to explore the recent advancements in nanotheranostics for cancer therapy and diagnosis. Then, it is attempted to present recent studies on nanotheranostics used as a contrast agent in various imaging modalities and a platform for cancer therapy.

## 1 INTRODUCTION

According to the estimates in 2018, there are about 18.1 million new cancer cases and 9.6 million cancer‐related deaths, with the most common and the main cause of death in men and women being lung and breast cancers, respectively [1]. Nanomedicines can overcome some limitations such as the multidrug resistance of cancer cells, the low solubility of hydrophobic anti‐cancer drugs, biocompatibility, and harmful radiation [2]. With the advances made in nanomedicine, theranostics is used as an effective approach for the diagnosis and treatment of malignant cancers at the cellular and molecular levels because of its high accuracy and reliability [3, 4]. In other words, nanoparticles can be used as diagnostic, therapeutic, and together as nanotheranostic agents. The rapid development of nanotheranostics can help in the early diagnosis of cancer and in therapies.

Various molecular imaging modalities including optical imaging (OI), positron emission tomography (PET), single‐photon emission computed tomography (SPECT), magnetic resonance imaging (MRI), ultrasound (US), computed tomography (CT), and X‐ray scatter imaging are used. Each of these techniques have advantages and limitations that need to be taken into account.

Consequently, routine therapeutic methods for cancer treatment are radiotherapy (RT), photodynamic therapy (PDT), and photothermal therapy (PTT).

**FIGURE 1:**
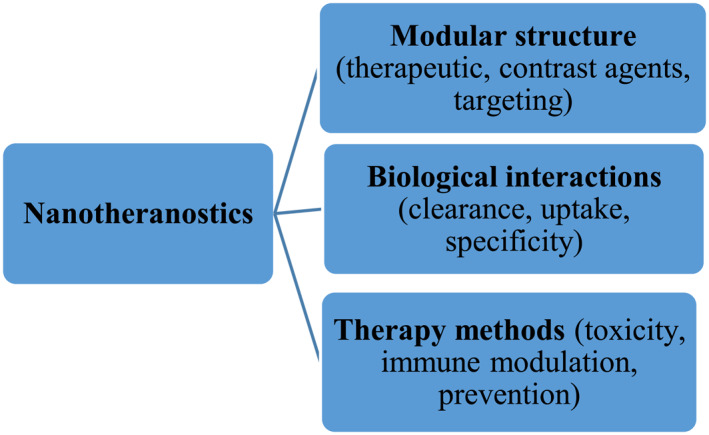
Properties of nanotheranostic

Recently, different kinds of nanotheranostic contrast agents like small molecules, peptides, aptamers, dendrimers, high‐molecular‐weight antibodies, and their combination with the various types of nanoparticles have been introduced [5]. As theranostic agents, nanoparticles have special advantages compared to small molecules [6] (Figure 1). Comparison of nanoparticles with small molecules such as iodinated molecules has revealed that nanoparticles have the advantages of long blood‐pool residence time and specific/targeted molecular imaging. For instance, their half‐life circulation is 15 h, whereas it is only some minutes for iodinated small molecules [7]. According to the previous studies [8–12], metal base nanoparticles could have a radiation sensitizer role. Moreover, the size and the shape of nanoparticles are of great significance. For instance, the smaller nanoparticle has the more concentration in the cell than the larger nanoparticles.

In addition, if the agent size is larger than 5 nm, renal clearance is slower than smaller agents, and if it is larger than 1 μm, they will not have renal clearance [13]. Comparison of spherical and cubic nanoparticles has indicated that spherical nanoparticles have a slow degradation as they have a lower contact surface for degradation relative to cubic nanoparticles [14].

The purpose of using molecular imaging nanoparticles is to reveal the smallest possible number of cancer cells before angiogenesis. Using therapeutic nanoparticles is to deliver the therapeutic agents to specific locations that reduces the off‐target toxicity of many drugs. Moreover, nanotheranostics are used because cancer theranostics reduce multi‐step procedures [15, 16, 17].

This review article emphasized the recent research advances on the application of nanotheranostics in molecular imaging and cancer treatments in the last few years. The challenges and future directions of theranostic agents were also summarized.

## 2 MOLECULAR IMAGING

In recent years, nanoparticles have been considered as a diagnostic contrast agent in molecular imaging.

In addition to the mentioned imaging modalities, multi‐modal imaging has been the focus of recent studies. This is due to each imaging modality has its advantages and limitations. For example, CT has high resolution and the possibility of three‐dimensional reconstruction, but its contrast is low for soft‐tissue discrimination. Using magnetic nanoparticles as a contrast agent, MRI with T_2_‐weight has high sensitivity and excellent soft‐tissue contrast. However, the intrinsic dark signals of contrast agents may be confused with other hypointense areas [18]. Hence, using multi‐modal imaging can overcome these problems.

## 3 NANOPARTICLES IN MR IMAGING

**FIGURE 2:**
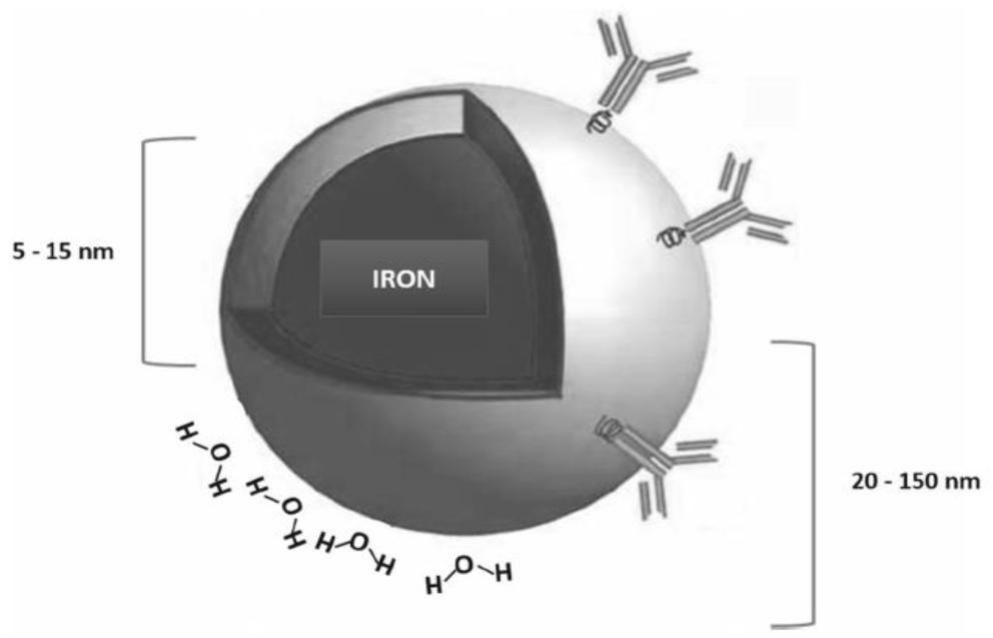
Schematic diagram of SPIONs [14]

It is notable that although MRI is suitable for soft tissue and has great spatial and temporal resolution, but it has low sensitivity and long imaging time. Therefore, using contrast agents can enhance its signal. Materials used for this purpose includes ferromagnetism (negative agents) and paramagnetic (positive agents). Superparamagnetic iron oxide nanoparticles (SPIONs) were the primary contrast agent for MR imaging [19, 20]. Figure 2 shows SPIONs with a core 5–15 nm radius and a total radius of the core with a shell and a water coat of 20 to 150 nm [14].

**FIGURE 3:**
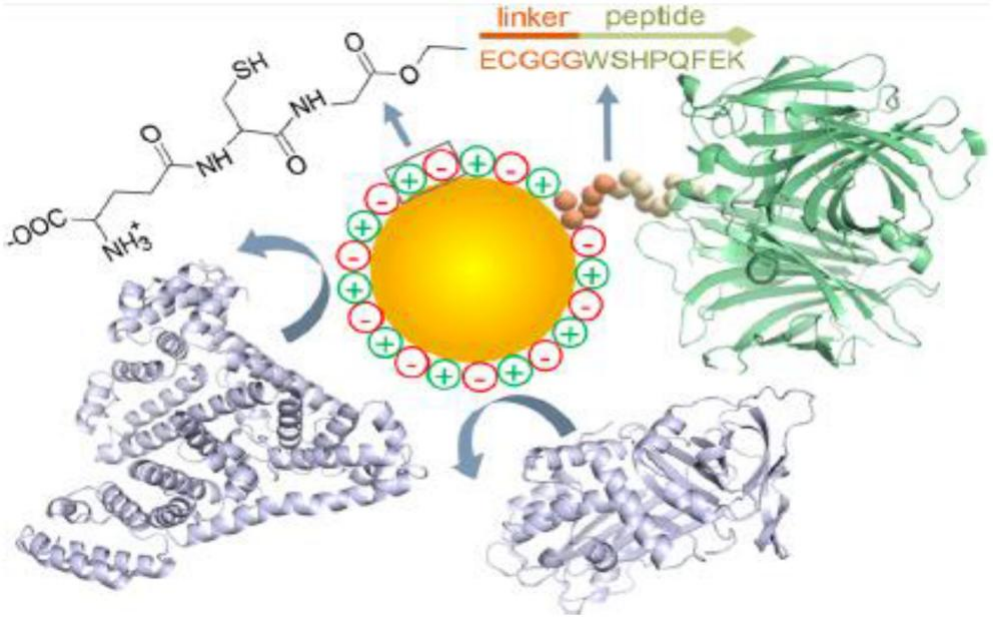
Schematic of AuGSH_zwt_ as an ultrasmall agent [40]

As a member of the ultrasmall SPIONs (USPIONs) family, ferumoxytol reduces T_1_ and T_2_* relaxation times [21]. It has an anti‐cancer therapeutic effect, and intravenous administration of ferumoxytol prevent metastasis to the liver [22]. Moreover, SPIONs in breast cancer can inhibit neovascularization and induce autophagy in endothelial progenitor cells [23, 24, 25]. High doses of SPIONs can damage the cytoskeleton and reduce cell proliferation rate [26]. It has been reported that if the concentration of Fe_3_O_4_, Al_2_O_3_, and TiO_2_ is less than 200 μg/ml, it has no measurable cytotoxicity effects [27]. Moreover, it has been shown that using SPIONs can lead to severe damage to DNA, protein, and inflammation [28, 29]. Gold nanoparticles do not have such cytotoxicity and can be used for MR, photoacoustic, fluorescence, and X‐ray scattering imaging [30, 31]. Moreover, when exposed to NIR laser light, they produce heat, making them suitable for photothermal therapy (PTT) of cancer [32, 33]. Consequently, several studies reported using AuNPs ultrasmall and nanoclusters coated with natural tripeptide glutathione (GSH) as MR imaging nanoprobe [8, 34, 35, 36, 37]. So, ultrasmall GSH‐coated AuNPs (AuGSH) in a size threshold of 2 nm, are not stable in biological fluids [38]. To overcome size‐dependent stability, a new ultrasmall AuNPs by substitution of the negatively charged GSH (GSH has a net charge of −1) ligand for its zwitterionic derivative, glutathione monoethyl ester was used. The new AuGSH_zwt_ has better colloidal stability and high resistance to serum protein interactions [39]. It was concluded that ultrasmall AuNPs including zwitterionic glutathione (AuGSH_zwt_) monoethyl ester surface coating have a higher resistance to aggregation (Figure 3). Indeed, they are effectively removed from the circulatory system owing to their small size and resistance to serum protein interactions. The obtained results indicated that optimizing the density onto the surface of ultrasmall AuNPs ligands is essential to enhance their performance [40] in imaging and in particular for cancer therapy.

The common commercially used contrast agent in MRI is gadolinium; however, given its low molecular weight, it has a short lifetime during blood circulation [41]. To resolve this problem, low molecular weight contrast agents can be conjugated to macromolecules like polysaccharides (pullulan) [42], synthetic polymers [43], and synthetic particles [44]. Nonetheless, gadolinium can end in a rare but severe nephrogenic fibrosis [45].

Manganese‐based contrast agents can be categorized into two groups: Mn^2+^ and manganese oxide nanoparticles (MnONs). Mn^2+^ has a short blood circulation time [46] and is toxic at high doses [47, 48], making it an imperfect contrast agent for MR imaging. MnONs have slight toxicity [49] and can lead to decreased hypoxia and improved treatment [50]. Nowadays, MnONs are more commonly used in the treatment and diagnosis compared to the gadolinium compound.

Generally speaking, in order to improve image sensitivity in MRI, different T_1_/T_2_‐weighted contrast agents based on gadolinium (Gd), manganese (Mn), and iron oxide nanoparticles (Fe_3_O_4_ NPs) have been developed. Gd has suffered from short blood circulation time and toxicity in vivo, which is potent to cause nephrogenic systemic fibrosis. Unfortunately, SPIONs, especially Fe_3_O_4_ NPs, have been somewhat limited in their clinical application because of their intrinsic dark signals and susceptibility artefacts in MRI, which means it is difficult to make a distinction between small early stage tumours and hypointense areas. Therefore, Mn‐based agents are considered ideal substitutes because of their bright signals and good biocompatibility [51].

Chen et al. [52] reported the use of transesterified oleic acid with N‐(trimethoxysilylpropyl) ethylenediamine triacetic acid (TETT) silane as water soluble accompanying MnO. Hu and colleagues [53] have coated polyvinylpyrrolidone (PVP) on MnO NPs (MnO@PVP NPs) for passing through the blood–brain barrier (BBB) and to gradually metabolize to other sites with blood flow.

Wang et al. in 2020 have examined Enolase 1 (ENO1)‐targeted SPIONs using the ENO1 antibody to evaluate pancreatic ductal adenocarcinomas (PDACs), which are the deadliest cancer with a 5‐year survival rate of 5% for all stages [1, 54]. According to their results, this nanoparticle increases the efficiency of early diagnosis of PDACs MR imaging in vitro and in vivo [55].

Studies have shown that epidermal growth factor receptor (EGFR) is involved in physiological processes like cell growth, proliferation, differentiation, and apoptosis. Moreover, it is involved in the formation, progression, metastasis, and angiogenesis of cancer [56, 57]. According to the clinical data, over 70% of malignant tumours have abnormal EGFR expression [58, 59]. In recent years, EGFR‐targeted imaging has been used as a non‐invasive method to evaluate EGFR expression levels [60]. For instance, a study was conducted in 2019 on SPIONs conjugated with anti‐EGFR. The authors used PLGA to encapsulate SPIONs to prevent biodegradation. The results showed that PLGA can increase the half‐life of nanoparticles and reduce side effects [61, 62]. Moreover, polyethylene glycol (PEG) binds to PLGA because of its beneficial effects [63, 64]. In this study, this nanoparticle is proposed for molecular imaging of MRI in EGFR diagnosis.

## 4 NANOPARTICLES IN SONOGRAPHY

Sonography is a real‐time non‐invasive method with high soft‐tissue image contrast using ultrasound. Contrast agents used in this modality are non‐microbubble including echogenic liposomes, perfluorocarbon nanodroplets, and solid nanoparticles and gas‐filled microbubble (a gaseous core such as air, nitrogen, perfluorocarbon, and sulphur hexafluoride that are surrounded by a shell of albumin, lipids, and synthetic polymer, or galactose with a diameter of 2–1 μm). Microbubble has high sensitivity and specificity. It is of note that although B‐mode ultrasound imaging is highly sensitive, it has low specificity for malignant lesions [65, 66]. Thus, combining it with targeted microbubbles increases its specificity. According to ultrasound studies, the accumulation of nanoparticles and the spread of drugs in tumours can be enhanced through cavitation. For instance, PEG‐PDLA (polyethylene glycol‐poly(D‐lactide) nanoparticles are used to overcome the aqueous solubility barriers paclitaxel under US guidance [67].

## 5 NANOPARTICLES IN COMPUTED TOMOGRAPHY

Another modality is CT imaging with advantages like high resolution, fast data collection time, deep tissue penetration, and ease of three‐dimensional tissue reconstruction. However, it has a low signal‐to‐noise ratio (SNR) and the limitation in the differentiation of soft tissues with the same density. Therefore, CT contrast media are applied to enhance soft tissue contrast [68]. Atoms like iodine, tungsten, and barium are usually used as contrast media in CT.

Recently, nano‐scale metal organic frameworks (NMOFs) due to the incorporation of high Z element and gold nanoparticles (AuNPs) due to the large X‐ray absorption coefficient are widely used as CT contrast media [5, 27]. Expected doses of CT contrast moieties can be condensed when NPs are used. For this reason, NPs have low number density, viscosity, and osmolality compared with same concentration of molecular contrast agents. Subsequently, by administration of nanoparticle CT contrast the imaging time and less renal toxicity will be expected [4].

## 6 NANOPARTICLES IN X‐RAY SCATTER IMAGING

X‐ray scatter imaging is based on the difference in X‐ray penetration intensity. This imaging modality has received great attention regarding the differences in the intensity of X‐ray penetration induced by the differences in thickness and tissue density [69]. According to the results of AuNPS studies, owing to their higher atomic number than iodine nanoparticles and lower toxicity, these particles are ideal contrast media for X‐ray scatter imaging [70, 71].

Rand et al. [72] used AuNPs coated with polyelectrolyte as a contrast media in X‐ray scattering imaging of hepatocellular carcinoma (HCC) cell pellets. They compared 10 and 50 nm AuNPs and showed that ultrasmall AuNPs have a higher sensitivity as a contrast media in X‐ray scatter imaging and are better in the diagnosis of small clusters of HCC than traditional X‐ray diffraction [72].

## 7 NANOPARTICLES IN OPTICAL IMAGING

Optical imaging is a non‐invasive method that allows visualizing and measuring different characteristics of a particular organ using various colours of light. In this modality, the penetration depth is low. A solution to deal with this issue is to use near‐infrared (NIR) light at 650–900 nm.

New optical imaging as molecular imaging modalities are endoscopy, optical coherence tomography (OCT), photoacoustic imaging (PAI), Raman spectroscopy, diffuse optical tomography (DOT), and supper‐resolution microscopy. Nowadays, using NPs have been considered in this modality. In a study in 2013, pyrene was conjugated on the shell of silica/poly(4‐vinyl benzyl chloride‐co‐pyrene‐1‐ylmethyl acrylate) core‐shell particles. The results indicated the high fluorescence intensity because of the presence of pyrene in the polymer shell [73].

The results of a study by Zhang et al. revealed that UiO‐66 (Universiteteti Oslo) is a metal organic framework made up of [Zr_6_O_4_ (OH)_4_] with 1,4‐benzodicarboxylic acid within 1,2‐dioleoyl‐sn‐glycero‐3‐phosphate (DOPA) lipid bilayer (DOPA‐LB) (UiO‐66@DOPA‐LB) has enhanced stability and impressive blood circulation time and higher accumulation in the tumour. Moreover, UiO‐66@DOPA‐LB labelled with a NIR dye, IRDye 800 nm CW, allowed imaging the breast cancer tumours and the early tumour detection [74].

Sokolov et al. [75] have coupled monoclonal anti‐EGFR antibodies to gold nanoparticles measuring 5 and 40 nm. Their results showed that gold nanoparticles with a size of 5 nm have strong infrared absorption and its photoacoustic signal is similar to 40 nm particles. Moreover, they showed that the particles as big as 5 nm have high penetration and clearance in vivo given their small size.

Using NIR‐emitting quantum dots (QDs) in cancer diagnosis has been also considered in this regard. NIR imaging has advantages like the lowest absorption in the tissue and the deepest tissue imaging. In 2018 Liu et al. [76] used carbon dots (CDs) as fluorescence nanomaterials. For the first time, this study presented the synthesis of highly luminescent CDs using folic acid (FA). They obtained fluorescence quantum yields (QY) of CDs higher than those of previous studies (94.5% in water). This QY was also higher than those of the organic fluorescent dyes.

## 8 NANOPARTICLES IN PET

Although PET imaging has high sensitivity and no limitation of depth penetration, but it lacks the anatomical information to detect molecular events. On the other hand, imaging modalities such as CT and MRI are combined with PET to overcome this limitation. Using multi‐modality imaging PET/CT and PET/MRI can detect the lesions/tumours from point of both functional and anatomical view.

Hajiramezanali et al. in 2019 [77] coated SPIONs with N,N,N‐trimethyl chitosan (TMC) and bombesin (BN) as targeting ligands and S‐2‐(4‐isothiocyanato benzyl)‐1,4,7,10‐tetraazacyclododecane tetraacetic acid (DOTA) as chelators and then labelled them by ^68^Ga. Their results indicated that the nanoparticle was suitable for PET/MRI for the diagnosis of prostate, breast, and lung cancers.

## 9 NANOPARTICLES IN SPECT

SPECT imaging is based on gamma rays detection with less sensitivity and spatial resolution than PET. However, based on reports, the micro‐SPECT spatial resolution used in the preclinical examination is higher than PET. In this modality, 99mTc imaging is the most useful for this purpose.

Zhao et al. [78] doped Au nanoparticle with ^199^Au and examined them using SPECT. According to their findings, when Au nanoparticles were doped with ^199^Au conjugated with D–Ala1–peptide T–amide, they could serve as a C–C chemokine receptor 5 (CCR5)‐targeted nanoprobe for the sensitive and specific detection of both triple‐negative breast cancer (TNBC) and its metastasis.

## 10 NANOPARTICLES IN MULTI‐MODAL IMAGING

As stated previously, owing to the limitations of each of the imaging modalities, nowadays multi‐modal imaging has received much attention. In this approach, Wei et al. in 2017 have examined the performance of NaGdF_4_@CaCO_3_‐PEG core‐shell nanoparticles in the diagnosis of prostate cancers. According to their results, under the acidic conditions of the tumour environment, the gradual dissolution of CaCO3 facilitates the collision of NaGdF_4_ with the surrounding liquid medium. This process leads to the enhancement of the comfort of the water proton and the MRI signal. Moreover, it creates CO_2_ bubbles form CaCO_3_ dissolution, creating strong echoes for ultrasound imaging [79].

In another study, porphyrin‐phospholipid (PoP), coating the core‐shell upconversion nanoparticle (UCNP), was used for lymph node mapping. The results indicated that PoP‐UCNPs can be computed using six techniques: near‐infrared (NIR), fluorescence (FL), NIR‐to‐NIR upconversion luminescence (UC), photoacoustic (PA), Cerenkov luminescence (CL), CT, and PET [80].

Fluorescence nanoparticles have been examined as imaging agents given the advantages of specificity betterment [81, 82], increasing circulation time, smart activation, and increase in signal intensity [81, 83]. Liu et al. have labelled SPIONs with Cyanine7.5 NHS ester and coated them with PEG by the immune system to reduce the effect of opsonization on circulation. In this study, first, U87 MG and two weeks later SPIONs were injected into mice. They found that brain tumour MR imaging signal was strong, and the fluorescence image of the major fluorescence intensity in the brains of the mice that received the substance showed higher than the control mice [84].

In this line another study evaluated the expression of B7‐H3 in normal breast cells, ductal carcinoma (DCIS) lesions in situ with low, intermediate, and high grade. Ultrasound as a molecular imaging modality was performed using B7‐H3 targeted microbubbles. The molecular photoacoustic and fluorescence imaging were performed together with the B7‐H3‐ICG antibody dye contrast agent. The results indicated that the expression level of B7‐H3 is grade dependent. Moreover, US, PA, and FL imaging in combination with B7‐H3 targeted contrast agents can diagnose DCIS in the murine model of breast cancer [85].

Zheng et al. tested AS1411‐Manganese‐Molybdenum disulphide quantum dots (Mn‐MoS_2_ QDs) for renal cell carcinoma [86]. Their results showed that AS1411‐Mn‐MoS_2_ QDs provide a new nanoprobe for MRI and fluorescence imaging and reported that it is a better MR contrast agent in renal cell carcinoma than Gd‐DTPA.

Because of increasing dispersibility and compatibility, lower toxicity, the possibility of conjugating with bioactive molecules, and increasing stability core‐shell nanoparticles can be used in biomedical applications [87, 88, 89, 90]. Contrast or dye agents can be attached to the core or shell. When in core‐shell substances like heparin, glucose, and steptavidin in core‐shell nanoparticles penetrate the shell and react with the nanosensor in the nucleus, they can generate a fluorescence signal [91]. However, these substances may end in destructive effects on the living system [92]; thus, degradable polymers are used to insulate these nanoparticles to avoid their accumulation in the body. For example, Aouidat et al. reported that gold gadolinium‐core‐shell nanoparticle is a diagnostic agent for evaluating hepatocytes in the liver [93].

## 11 NANOPARTICLES IN RADIATION THERAPY

In addition to diagnostic applications, nanoparticles can also be used in therapies. An important task in current cancer therapies is the lack of selectively, which to damage in healthy tissues. Therefore, researcher attempts have focussed on new targeting agents to minimize healthy tissue damage.

For instance, they can encapsulate pharmaceuticals like doxorubicin (DOX) and release them at targeted sites, and thus reduce systemic toxicity and enhance pharmacokinetic profile [6]. Indeed, nanoparticles offer the ability to transport therapeutic agents to specific sites of a tumour, leading to a reduction in the off‐target toxicity of many drugs. This is seen in chemotherapy, which happens with serious side effects [8].

In a study the fluorescence lifetime imaging microscopy (FLIM) imaging technique was applied in monitoring the dynamic change of DOX fluorescence lifetime in intercellular environments. In this study, poly(allylamine)‐citraconic anhydride/doxorubicin (PAH‐Cit/DOX) nanoparticles were used as the nanodrug system. DOX was released from the nanoparticle by acidic pH triggering in endosomes and lysosomes using this nanoparticle. Also, FLIM was used for monitoring the release and subcellular distribution of DOX possible. In this study, after the Phasor‐FLIM analysis, the fluorescence lifetimes of DOX were separated into four segments in the phasor plot. It was shown that the four parts of FLIM images mainly located in cell membrane, cytoplasm, nucleus membrane and nucleus respectively. And the average lifetimes in these four parts were 4.46, 3.16, 2.34, and 1.52 ns. The results meant that DOX had a similar fluorescence lifetime in one of these cellular compartments. When coming to another compartment, its lifetime changed. The lifetime difference in these compartments might be attributed to the change of physicochemical environments and the drug release from the nanocarrier. Indeed Phasor‐FLIM analysis was successfully separated subcellular compartments with DOX under a different releasing rates, based on the difference of DOX fluorescence lifetime [94].

In 2016, polymeric doxorubicin (pDOX) was loaded into an injectable nanoparticle generator (iNPG). The results indicated that iNPG‐pDOX was more effective in breast metastatic cancers compared to DOX alone [95].

Adjuvant drug labelled liposome‐and lipid‐based nanoparticles can be covalently attached to the cell surface to treat adoptive T‐cell therapy and thus reduce tumour burden [96]. According to a study, PEG‐PLGA nanoparticles encapsulated with green indocyanine (ICG) and TLR7 agonist R837 can produce tumour‐related antigens during photothermal therapy (PTT). Moreover, its combined treatment with anti‐CTLA4 antibodies inhibited metastasis in the 4T1 orthotopic model [97]. Xiaoran et al. (2017) have used hollow‐structured CuS@Cu_2_S@Au nanohybrid to increase photothermal treatment and photoswitchable targeting theranostic. In this study, the treatment efficiency increased after loading doxorubicin, as photodynamic therapy in combination with chemotherapy increases tumour treatment effectiveness [98]. They compared this nanoparticle with a plasmonic metal core/semiconductor shell and stated that this nanoparticle beside increasing the efficiency of photothermal conversion (808 nm laser radiation), provides large cavities and a mesoporous shell for loading and releasing drugs [99].

In 2019, it was found that chitosan‐coated poly(d,l‐lactide‐co‐glycolide) (PLGA) nanoparticles could be used as a drug delivery system for diosmin orally [100]. Diosmin properties are anti‐inflammatory properties, free radical scavenging [101], and anti‐ulcer activities [102]. However, this drug has little solubility and needs high oral doses [103]. According to the results, chitosan‐coated PLGA can increase the residence time of the drug at the delivery site, improve bioavailability, lower drug dose, lower dosing frequency, and reduce side effects [104].

An et al [105] used ester poly(β‐amino) core and hyaluronic acid shell to transfer doxorubicin to cells to overcome the drug resistance of chemotherapy drugs in examining breast cancer. According to their study, hyaluronic acid could improve the transmission of doxorubicin. In this study, using this nanoparticle increased the uptake and apoptosis. Wu et al. [106] created PLGA‐alginate core‐shell particles. Although this nanoparticle showed lag phase similar to PLGA microparticles in their release kinetics, they reduced initial burst release but the drug release was slower than PLGA microdistricts because the alginate shell was a barrier to retard the diffusion of the drug.

## 12 THERANOSTIC NANOPARTICLES

Nanomedicine theranostics are the nanoparticles that can act as both diagnostic and therapeutic agents. Combining the diagnosis and treatment of cancer theranostics has received special attention because of the reduction of multi‐step procedures [16,17] and the use of patient‐specific test results to tailor a treatment regimen producing improved outcomes, reduced costs, and fewer side effects [107]. Nanotherapeutics should have pharmacological activity, stay in the body for a long time, and create special side effect during the treatment process. Among the advantages that ideal theranostics nanoparticles should have the ability to highly selective accumulation, the ability to deliver an effective therapeutic action selectively, safe and nontoxic are vital. [108, 109].

Nanodiagnostics should not be pharmacologically or toxicologically active and instead must have biodegradation and rapid elimination. Moreover, the occurrence and severity of its side effect should be minimal [53].

Nowadays, gold nanoparticles are used as diagnostic agents, and theranostics because of optochemical properties, biological efficiency as a biomarker, and high absorption coefficient of X‐rays [110, 111].

Nanoscale metal‐organic frameworks (NMOFs) have received great attention as the contrast agent for imaging and treatment because of their diversity of structure, high porosity, multi‐functionality, and biocompatibility. Given the EPR effect, NMOFs can accumulate in the tumour, which is passive targeting. Meanwhile, the active targeting is attained by adding ligands to the surface of NMOFs. According to the results of a study, long‐term toxicity and biosafety related to NMOFs should be evaluated in this regard. Furthermore, NMOFs platforms have shown anti‐cancer effects in animal models, but their function in the human body should be examined. Moreover, they stated that although ligands can bind to NMOFs, it is difficult to detect healthy and cancerous cells. Besides, building NMOFs needs a complex chemical synthesis [112].

Huang et al. [113] studied the multi‐functional chitosan modified Gd_2_O_3_: Yb^3+^, Er^3+^@nSiO_2_@mSiO_2_ core/shell nanoparticle. According to this study, Gd_2_O_3_, Er^3+^, Yb^3+^ core can be effective contrast agent for MRI, and showing red signals under the 980 nm laser excitation. In addition, the study showed that using folic acid with chitosan and doxorubicin load results in higher cytotoxicity for Hela cells under in vitro conditions.

In a study, ultrasmall AuNPs were investigated. The study examined the methods of size control and surface functionalization of ultrasmall AuNPs as well as the studies conducted in the field of imaging and cancer treatment. The results indicated that despite its diagnostic and therapeutic applications, more studies are needed to optimization of gold nanoparticles in clinical applications [69].

Mirković et al. have been used 99mTc‐Fe_3_O_4_‐HEDP‐MNPS nanoprobe in ex‐vivo biodistribution studies and they showed significant uptake in the liver and spleen in healthy Wistar rats after intravenous administration [114]. Moreover, the results of scintigraphy studies indicated high in vivo stability. Based on this study, this nanoprobe has the potential to be used as a theranostic.

## 13 CONCLUSIONS AND FUTURE PERSPECTIVES

Nanotheranostics is of great importance in early diagnosis and therapy of cancers. Despite the widespread use of nanotheranostics, several factors such as their pharmacokinetic properties, toxicity, biodegradation, and elimination have to be given special attention in the case of using nanoparticles. Considering these points, to conduct clinical studies, it is necessary to understand the advantages and limitations of nanoparticles and their characteristics in various imaging modalities and therapeutics. Table 1 shows different nanotheranostics and their applications in imaging modalities and therapeutics. In this way, it is possible to use them with the right and comprehensive insight into their clinical applications. If this happens, by proper selection and use of nanoparticles, this method can lead to a revolution in tumours diagnosis and treatment. Among the mentioned nanoparticles, AuNPs are candidates for various modalities because of their low inherent toxicity, high surface area, ability to functionalize easily with biomolecules, outstanding radio enhancer, and their enhanced optical properties in both diagnosis and treatment. However, their poor clearance, skin discolouration, and cost are limitations. Recently, researchers found iodine compounds as an alternative to gold. Iodine is a colourless compound, has good X‐ray absorption and better clearance pathways, and is relatively low cost.

**TABLE 1:** Applications of nanotheranostics in imaging modalities and therapeutics

Nanoparticles	Imaging Methods	Cancer Therapy	References
Conjugating PEPHC1 to PEGylated SPIONs	MRI/fluorescence image	‐	[84]
AuNPs	MRI/photoacoustic/fluorescence/X‐ray scattering imaging	PTT	[30, 31, 32, 33, 72]
Mn_3_O_4_	MRI	Chemotherapy/PDT/PTT	[51]
Monodisperse silica−polymer core−shell particle	Fluorescence imaging	‐	[73]
^68^Ga‐DOTA‐BN‐TMC‐MNPs	PET/MRI	‐	[77]
Hyaluronic acid‐coated poly (β‐amino) ester nanoparticles	‐	Chemotherapy	[105]
NaGdF_4_@CaCO_3_‐PEG core‐shell nanoparticles	MRI/ultrasound imaging	‐	[79]
PoP‐UCNPs	Near‐infrared/fluorescence/NIR‐to‐NIR upconversion luminescence/photoacoustic Cerenkov luminescence/CT/PET	‐	[80]
Mn‐MoS_2_ QDs	MRI/fluorescence imaging	‐	[86]
UiO‐66@DOPA‐LB	OI	‐	[74]
ENO1‐targeted SPIONs	MRI	‐	[53]
iNPG‐pDOX	‐	Chemotherapy	[95]
CuS@Cu_2_S@Au nanohybrid	‐	PTT/chemotherapy	[98]
Gd_2_O_3_:Yb^3+^, Er^3+^@nSiO_2_@mSiO_2_ core/shell nanoparticle	MRI/OI	Chemotherapy	[113]

Overall, based on the literature here, further studies are needed to investigate the properties and pharmacokinetics of nanotheranostics in various models in vitro and in vivo for their clinical applications.
